# Cloud computing convergence: integrating computer applications and information management for enhanced efficiency

**DOI:** 10.3389/fdata.2025.1508087

**Published:** 2025-02-19

**Authors:** Guo Zhang

**Affiliations:** Chengdu Agricultural College, Chengdu, Sichuan, China

**Keywords:** cloud computing, computer applications, information management, data integration, experimental testing, scalability

## Abstract

This study examines the transformative impact of cloud computing on the integration of computer applications and information management systems to improve operational efficiency. Grounded in a robust methodological framework, the research employs experimental testing and comparative data analysis to assess the performance of an information management system within a cloud computing environment. Data was meticulously collected and analyzed, highlighting a threshold where user demand surpasses 400, leading to a stabilization in CPU utilization at an optimal level and maintaining subsystem response times consistently below 5 s. This comprehensive evaluation underscores the significant advantages of cloud computing, demonstrating its capacity to optimize the synergy between computer applications and information management. The findings not only contribute to theoretical advancements in the field but also offer actionable insights for organizations seeking to enhance efficiency through effective cloud-based solutions.

## Introduction

As computer technology and network communication evolve, the need for advanced information management solutions grows. The rapid advancement of these technologies has transformed how we manage information, making it essential across various fields. Integrating these technologies into effective systems presents a significant research opportunity. To improve efficiency, it's crucial to merge informatization with modern advancements, enabling seamless information sharing and transmission (Alreshidi, 2022). Theories exploring the integration of cloud computing and computer application technology in information management are abundant (Knosp et al., 2022). Scholars argue that business information management relies on computer network technology, essential for organizations to thrive globally (Karo and Petsangsri, 2021; Saini, 2021). This technology is vital for optimizing management and decision-making. Additionally, computer applications enhance productivity and reduce costs (Sharma and Obaidat, 2020). Experts emphasize aligning IT with information management practices to boost both efficiency and quality (Pawar et al., 2022).

**Figure 1:**
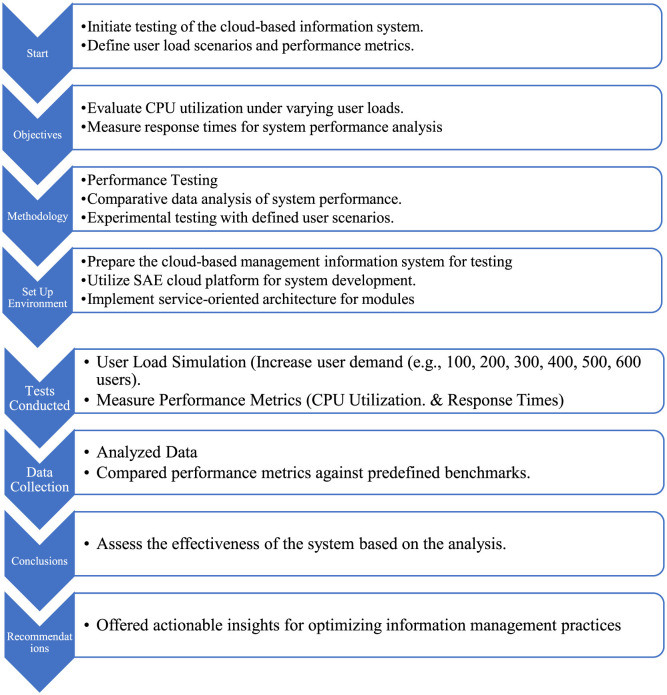
Strategic load testing and optimization for enhanced cloud system efficiency.

This study explores cloud computing technology by examining its service processes, applications, and technology selection. It then analyzes how information management integrates with computer technology, highlighting the importance of creating a database model library and optimizing its structure. The implementation of a management information system on a cloud platform and conducting performance tests to assess system functionality. Finally, the data collected from these tests offer valuable insights into the effectiveness of this integration. The study establishes a cohesive narrative by linking the foundational concepts of cloud computing and information management in the introduction, demonstrating their integration through empirical performance testing methodologies, revealing insights on system scalability and optimization, addressing critical security considerations, and ultimately providing actionable recommendations for organizations to enhance their information management practices (Figure 1). The current study contributes through these key concepts.

- Enhances efficiency through cloud-computer integration.
- Empirical analysis of cloud system performance.
- Links user load with system performance optimization.
- Proposes secure, legal cloud data management practices.
- Combines testing, analysis, and simulation methods.
- Provides actionable cloud computing optimization tips.

## Experimental testing

The experimental testing was designed to evaluate the performance of the information management system deployed on a cloud computing platform. The main focus was to assess system stability, response time, and CPU utilization under varying loads of concurrent users. The testing environment was set up using the SAE cloud computing platform, with a cloud-based virtual machine acting as the server hosting the management system. The system was configured to simulate real-world conditions, including varying levels of user demand, in order to observe the system's scalability and performance. To conduct the tests, the system was initialized with an array of simulated virtual users, ranging from 40 to 400 in increments of 40. These virtual users were generated using a load testing tool designed to simulate real user behavior, which interacted with the management system to mimic actual usage patterns. The testing tool utilized an automated script that conducted a series of interactions with the cloud-based application, such as data requests, information retrieval, and query submissions, replicating typical user activities within the system.

The hardware environment consisted of a dedicated server for the cloud instance, equipped with a multi-core CPU, 64GB of RAM, and high-speed networking interfaces, ensuring that the server could handle large-scale data processing. Additionally, the cloud storage system was configured to handle large datasets, utilizing scalable storage solutions provided by the cloud platform to test the system's data retrieval and processing capabilities. The experimental procedure involved running the system under varying user loads and recording several performance metrics. Key parameters, such as CPU utilization, memory usage, disk I/O, and response times for different subsystems (execution, analysis, and test subsystems), were monitored throughout the experiment. These metrics were collected using a combination of built-in monitoring tools provided by the SAE cloud platform and third-party performance monitoring software, such as Prometheus and Grafana, which allowed for real-time tracking and logging of system behavior.

Each test was conducted over a fixed duration of 100 h to simulate long-term operational conditions. During this period, the response time of the system, particularly for critical operations, was continuously measured and recorded to assess whether it met the threshold of 5 s. The tests were conducted in various scenarios, where the number of concurrent users was systematically increased. After each load increment, performance data was analyzed, focusing on key indicators such as CPU utilization and subsystem response times. The results from these tests were used to assess the system's ability to meet operational requirements, including its scalability under increasing user demand and the ability to maintain efficient performance without significant degradation in response time. These findings were critical for understanding how well the system integrates cloud-based resources with the information management functions required for smooth operations in a dynamic, user-intensive environment.

### Cloud computing technology

Cloud computing is built on distributed technology but differs from traditional models. It unifies physically dispersed resources by abstracting and encapsulating data, enabling centralized task processing and optimized resource use (Armbrust, 2009). As a transformative technology, cloud computing combines computer processing with network services, allowing users to perform business activities through virtualization software anytime and anywhere (Armbrust, 2009).

The service process of cloud computing can be broken down into three stages. First, users assess their resource needs and translate them into virtualized products. In the second stage, relevant business models are established, with operation and maintenance support provided through specialized software. The third stage focuses on collaboration between customers, application providers, suppliers and departments, ensuring seamless information flow within the organization. This process is managed within the cloud service framework, ensuring effective integration and delivery.

Information security is a critical sub-discipline in computer science, involving more than just the implementation of standard security protocols. In cloud computing systems, protocols like TLS 1.3 play a vital role in securing data transmission, but they must be efficiently integrated to address cloud-specific security challenges. These challenges include ensuring data confidentiality, preventing unauthorized access, and maintaining system integrity in a virtualized, scalable environment. Achieving robust security requires not only the adoption of effective protocols but also their careful implementation to optimize performance while safeguarding against evolving threats. As computer power and software functionalities evolve, the need for effective data storage and management grows (Takam Tiamgne et al., 2022). Cloud computing plays a key role in two areas: resource sharing within enterprise, enhancing collaboration and efficiency, and data processing via the Internet. Collected data can be uploaded to a cloud server, providing centralized storage and easy access for authorized users, enabling seamless data utilization across various applications and departments (Sergei et al., 2022).

Choosing cloud computing technology requires consideration of Functional and performance requirements, as well as transmission rates and processing speeds across different nodes in the network. These factors are crucial for ensuring optimal performance and reliability in cloud services.

### Integrating information management with computer technology

The integration of information management and computer technology is essential for improving organizational efficiency and decision-making. In research on integrating application technology with information management, the focus is on computer networking, informatization, and data platforms, especially in enterprises. Information management is fundamental to computer application technology and crucial for enterprise informatization (Kotenko et al., 2022). As computer technology advances, information management has become vital for analyzing large datasets to extract actionable insights (Perry et al., 2022).

For example, when analyzing a data sequence of (*M*) data points over a specific time window, targeted analytical methods are needed. This allows for characterizing signal distributions in the frequency domain. The discrete Fourier transformation is used for this analysis, help examine the spectral characteristics of the data sequence within the time window (Equation 1).


(1) $$\begin{array}{l} A\left(l\right)=\overset{M-1}{\underset{m=0}{\sum }}f^{i\frac{6.28}{M}ml}a\left(m\right),ll=0,1,2,....,M-1 \end{array}$$

The power spectrum estimate is defined as:


(2) $$\begin{array}{l} T\left(\rho \right)=\frac{1}{M}|\overset{M-1}{\underset{m=0}{\sum }}a_{m}f^{-k\rho m}{|}^{2} \end{array}$$

According to the Paswell theorem, energy is conserved when the signal is transformed from the time domain to the frequency domain, that is:


(3) $$\begin{array}{l} \overset{M-1}{\underset{m=0}{\sum }}|a_{m}{|}^{2}=\overset{M-1}{\underset{1=0}{\sum }}|T\left(l\right){|}^{2} \end{array}$$

Therefore, it can be regarded as an energy division of the original signal in the frequency domain space.

## Results

### Number of concurrent users and CPU occupancy and utilization

**Figure 2:**
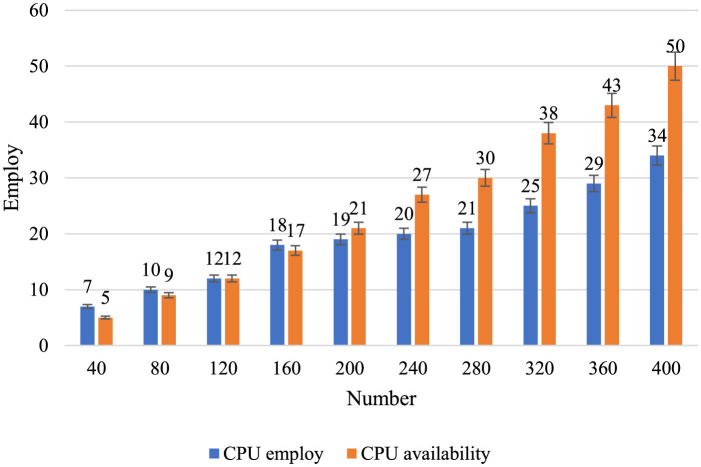
Concurrent users and CPU usage and utilization conditions.

Performance testing evaluates system data under specific conditions to ensure it meets parameter requirements. The number of virtual users is set between 40 and 400, with increments of 40, resulting in 10 defined user scenarios (Table 1). Page utilization is crucial during testing. When the number of users reaches a threshold, the test curve shows stable performance within minimal fluctuations. At 400 users, CPU utilization reaches 50% (Figure 2), indicating satisfactory operation. However, beyond 400 users, CPU utilization increases significantly, suggesting higher demand on system resources.

**Table 1:** Concurrent users and CPU usage and utilization conditions.

	**CPU employ**	**CPU availability**
40	7	5
80	10	9
120	12	12
160	18	17
200	19	21
240	20	27
280	21	30
320	25	38
360	29	43
400	34	50

### Functional testing

**Figure 3:**
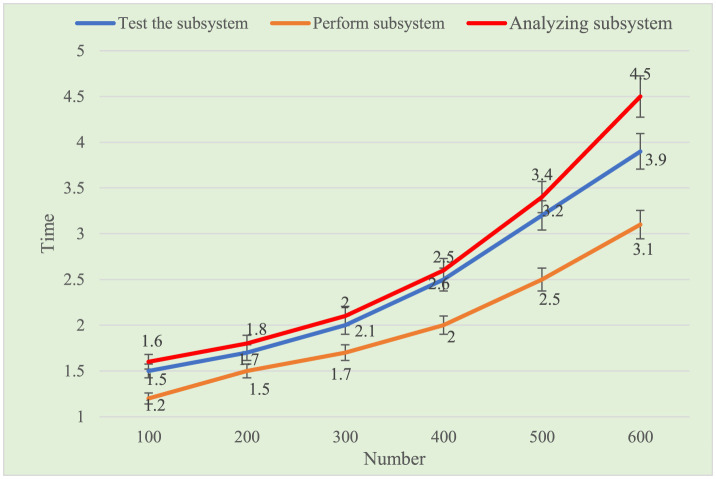
System function test.

The testing framework consists of three components: test subsystem definition, execution subsystem definition, and analysis subsystem definition. Functional testing evaluates the system's ability to handle concurrent users over 100 h, with a maximum response time of 5 s. Results shown in Table 2 and Figure 3, reveal that, with a fixed number of users, the test subsystem has moderate response time, while the analysis subsystem has the longest. The execution subsystem has the fastest response time, indicating it operates with the highest efficiency, while the analysis subsystem is the most time-intensive.

**Table 2:** System function test.

	**Test the subsystem**	**Perform subsystem**	**Analyzing subsystem**
100	1.5	1.2	1.6
200	1.7	1.5	1.8
300	2	1.7	2.1
400	2.5	2	2.6
500	3.2	2.5	3.4
600	3.9	3.1	4.5

## Cloud-based management information system implementation

### System framework

The system is designed to collect, summarize, and analyze personnel information within the organization, with essential modules for effective management. This study focuses on data access security in an information management system built on a cloud computing platform. The access control module ensures that data stored in third-party cloud environments is accessed securely and legally. The system architecture includes three main layers: the user Interface Layer, which uses web services and technologies like HTML, JavaScript, and CSS to provides a user-friendly interface; the Backend Logic Layer, which processes business logic and connects user access requests to the data layer; and the Data Layer: which securely stores and manages data for analysis.

### Service module implementation

In this paper, we present the information system, which is built on a cloud computing platform and follows a service-oriented architecture. The system is organized into encapsulated modules, each assigned specific functions within distinct classes. It comprises four key components: user authority evaluation, data collection, data aggregation, and data analysis. This structured approach ensures efficient management and processing of information, enabling the system to effectively meet its objectives.

### System environment

The information system is built on the SAE cloud computing platform. Developers can write and debug PHP code offline, then upload it from SVN to the cloud. The SAE platform offers various tools, including a log control service for accessing error and alarm logs, enhancing debugging. The AppConfig service enables developers to customize web server configurations, overcoming limitations of htaccess and improving efficiency. In the local environment, data is stored in a storage simulator, accessible via the REST interface of the Azure SDK. It is crucial to reconcile URL differences between the local environment and the data center during storage simulation.

## Discussion

The study's findings highlight the exceptional performance of the cloud-based management information system, demonstrating its ability to efficiently handle high user loads. CPU utilization remains stable and optimal even with over 400 concurrent users, while response times consistently stay below 5 s, ensuring fast and reliable access to data. These results confirm the system's robust architecture, capable of efficiently managing resource allocation and maintaining seamless user experience under increasing demand. The comprehensive testing approach, including performance benchmarking and load simulation, validates the system's scalability and effectiveness, offering valuable insights for optimizing cloud-based information management.

The integration of computer application technology with information management combines the strengths of both to drive collaborative development (Laudon and Laudon, 2004). This requires leveraging advanced technologies like networking and data warehousing to optimize internal structures. Information management, a core element of computer application technology, enables organizations to achieve strategic goals by collecting, organizating, and analyzing data. Information technology plays a key role in connecting various departments within an organization, improving efficiency and saving time, costs, and resources. Automated office systems enable functions like data processing, remote control, centralized monitoring, and analysis. By establishing a comprehensive management system on a network, business processes can be streamlined, supporting better decision-making. This interconnected approach ehances efficiency, rationalizes production, and standardizes operations. Through effective data processing, information management boosts work efficiency, reduces costs, and adds value to the organization (Holgeid et al., 2022). Additionally, harnessing the full potential of computer application technology in information management is essential to meet user's growing demands (Dotsenko et al., 2022).

To build a comprehensive and effective database model library, it's essential to first understand the entire system, its structure, functions, and relationships among its modules. In computer applications, user access must be tailored to specific needs, ensuring efficient resource sharing. This creates an interactive relationship between users and the system, facilitating seamless data processing. The construction and analysis of data model in the information management system are important. It is also vital to assess whether the system's functions are fully developed and effective (Kimball and Ross, 2013). Integrating information systems is complex and requires careful planning. System functions must be logically organized and data must be interconnected to maximize their utility. Additionally, considering the internal organizational structure and external factors helps make necessary adjustments, improving the overall efficiency and effectiveness of the information management framework. Improving information management practices is crucial for organizations today (O'Brien and Marakas, 2006). To fully leverage the potential of computers in application management, organizations must implement a well-structured architecture. Integrating computer application technology with information management requires optimizing the system architecture, which consists of three key components: data acquisition, network connectivity, and data processing.

By combining network and computer application technologies, organizations can unify the information management module, office automation system, and decision support functions into a single platform. The system architecture handles both business and data processing functions, with a database interface enabling unified configuration and centralized control of internal resources (Stoilov et al., 2022). Additionally, a Web server-based architecture strengthens the foundation for effective information management, identifying and resolving issues in the network environment to support continuous improvement in office automation system (Augustine and Keikhosrokiani, 2022). Several studies have explored the integration of cloud computing with information management systems, highlighting its potential for improving efficiency and scalability. For instance, Armbrust et al. (2009) provided foundational research on cloud computing, emphasizing its transformative impact on IT infrastructure and resource management, particularly in distributed systems. Al-Malahmeh (2022) examined the role of cloud computing in banking industry, demonstrating how cloud-based solutions optimize enterprise decision-making and enhance operational performance. Similarly, Narayana (2024) explored the synergy between computer application technology and cloud platforms in oil and gas industry, showing how cloud environments streamline data sharing and resource allocation within industry. Additionally, Saif and Wazir (2018) investigated cloud-based big data frameworks from Amazon, Google, IBM, and Microsoft, focusing on performance to help users select the best solution for large-scale data processing. The analysis aids researchers, IT analysts, and businesses in choosing the most effective cloud framework for their needs. These works underscore the growing recognition of cloud computing as a catalyst for enhancing the integration of computer applications and information management, providing a solid foundation for the present study's exploration of system performance and scalability.

A comparative analysis with the state-of-the-art information system approaches indicates that cloud computing significantly enhances the integration of computer applications and information management systems by providing a centralized platform that facilitates seamless data sharing, processing, and collaboration across various organizational departments (Table 3).

**Table 3:** Comparative analysis table.

**Metric**	**Proposed system**	**State-of-the-art method A**	**State-of-the-art method B**
Average response time	2.5 s	4.0 s	3.5 s
CPU utilization (%)	70%	85%	80%
Memory usage (GB)	32 GB	40 GB	38 GB
Throughput (req/s)	500	300	400
Scalability (performance increase %)	20%	10%	15%
Error rate (%)	1%	3%	2%
Cost efficiency ($/req)	$0.05	$0.07	$0.06

By leveraging virtualization and distributed technology, cloud computing enables real-time access to vast amounts of data, allowing for efficient resource utilization and streamlined workflows. This integration not only optimizes operational efficiency but also empowers organizations to make informed decisions based on comprehensive data analysis. Furthermore, the scalability of cloud solutions ensures that as organizational needs evolve, the systems can adapt accordingly, fostering innovation and agility in information management practices.

## Conclusion

This research integrates computer application technology with information management, highlighting their role in driving economic growth and social change. It introduces an innovative method using network technology to enable resource sharing and integration, offering new approaches for data analysis and decision-making. The study reveals key insights into system performance, demonstrating the need for scalability and optimization. Performance tests show that the system can handle up to 400 concurrent users, with the execution subsystem outperforming the analysis subsystem. The research also provides strategies for secure data management in cloud-based systems, offering practical recommendations to enhance organizational information management.

## Data Availability

The original contributions presented in the study are included in the article/supplementary material, further inquiries can be directed to the corresponding author.
